# Craniofacial Laterality Discrimination and Psychological Factors in Women with Fibromyalgia: A Cross-Sectional Study

**DOI:** 10.3390/healthcare14162636

**Published:** 2026-08-20

**Authors:** Víctor Riquelme-Aguado, Javier Bravo-Aparicio, Iria Trillo-Charlín, Hector Beltran-Alacreu, Stella Maris Gómez-Sánchez, Antonio Gil-Crujera

**Affiliations:** 1 Department of Basic Health Sciences, Rey Juan Carlos University, 28032 Madrid, Spain; stella.gomez@urjc.es (S.M.G.-S.); antonio.gil@urjc.es (A.G.-C.); 2 Research Group GAMDES, Department of Basic Health Sciences, Universidad Rey Juan Carlos, 28922 Alcorcón, Spain; 3 Pain, Mental Health, Exercise and Technology Research Group (PAIN+MET), Faculty of Physiotherapy and Nursing, Universidad de Castilla-La Mancha, 45071 Toledo, Spain; javier.bravo@uclm.es (J.B.-A.); hector.beltran@uclm.es (H.B.-A.); 4 Toledo Physiotherapy Research Group (GIFTO), Health Research Institute of Castilla-La Mancha (IDISCAM), 45004 Toledo, Spain; iria.trillo@uclm.es; 5 Toledo Physiotherapy Research Group (GIFTO), Faculty of Physical Therapy and Nursing, University of Castilla-La Mancha, 45004 Toledo, Spain

**Keywords:** fibromyalgia, laterality discrimination, motor imagery, body esteem, anxiety, kinesiophobia

## Abstract

**Background:** Fibromyalgia (FM) is a multidimensional chronic pain condition associated with alterations in sensorimotor processing, body perception, and psychological functioning. Although laterality discrimination impairments have been described for the limbs, evidence regarding craniofacial regions remains limited. This study compared mandibular and lingual laterality discrimination, body esteem, state anxiety, and kinesiophobia between women with FM and healthy controls and examined their associations with clinical variables. **Methods:** A cross-sectional study included 60 women, 30 with FM and 30 healthy controls. Mandibular and lingual laterality discrimination were assessed using a digital image-recognition task that recorded accuracy and reaction time. Body esteem, state anxiety, kinesiophobia, pain intensity, and FM impact were also evaluated. Between-group comparisons and correlation analyses were performed. **Results:** In unadjusted analyses, women with FM showed lower mandibular laterality accuracy and longer reaction times during the lingual task; however, these differences did not remain statistically significant after correction for multiple comparisons. Women with FM showed significantly higher state anxiety and kinesiophobia and lower body esteem than healthy controls, with these differences remaining significant after multiplicity adjustment. **Conclusions:** Women with FM showed marked psychological and body-related differences compared with healthy controls. Between-group differences in craniofacial laterality outcomes were observed in unadjusted analyses, although they did not remain statistically significant after correction for multiple comparisons.

## 1. Introduction

Fibromyalgia is a chronic primary widespread pain condition characterized by persistent diffuse pain, commonly accompanied by fatigue, sleep disturbance, cognitive difficulties, and emotional or functional impairment [1,2,3,4].

Beyond its physical manifestations, FM is associated with several psychological factors that may contribute to symptom severity and disability. Among these, fear of movement (kinesiophobia) has received considerable attention. Higher levels of kinesiophobia have been associated with greater pain intensity, increased disease impact, and higher levels of depressive symptoms in individuals with FM [5]. Furthermore, psychiatric comorbidities are highly prevalent in this population, with anxiety and depression affecting approximately 50.8% and 46.6% of patients, respectively [6]. Negative affective states have been linked to greater pain intensity, functional limitations, fatigue, cognitive dysfunction, sleep disturbances, and poorer quality of life [7,8]. In addition, anxiety has been associated with heightened pain perception and increased symptom somatization in patients with FM symptoms [9].

The persistent interaction between pain, emotional distress, and functional limitations may also influence how individuals perceive their own bodies. Previous studies have reported alterations in body image and lower body-related self-evaluation in women with FM compared with healthy individuals [10,11]. These alterations have been associated with poorer mental health, lower vitality, and reduced quality of life [12,13].

While body image reflects the cognitive and emotional perception of the body, body schema refers to the sensorimotor representation of the body used for movement and interaction with the environment. Body schema is influenced by proprioceptive information (kinesthesia, sense of force, sense of effort, and sense of balance), exteroception (tactile, visual, auditory, olfactory, and gustatory stimuli), and interoception (the perception of the physiological state of the body) [14].

Laterality discrimination tasks have been widely used as indirect behavioral measures potentially related to body-related sensorimotor representations and implicit motor imagery processes. These tasks require individuals to determine whether a presented body part corresponds to the left or right side of the body by mentally rotating its internal representation [15]. Laterality discrimination impairments have been reported in several chronic pain conditions. Although evidence in FM remains limited, existing studies suggest that these patients show reduced accuracy and slower performance during laterality discrimination tasks compared with healthy controls [10,16].

Orofacial pain represents a clinically relevant condition with a substantial population burden. Recent meta-analytic evidence has reported pooled prevalence estimates of approximately 20.6% for myofascial orofacial pain, 12.0% for combined myofascial and temporomandibular joint pain, and 9.5% for temporomandibular joint pain alone. Orofacial pain also frequently overlaps with other chronic pain conditions [17]. Recent evidence suggests that irritable bowel syndrome is associated with an increased risk of both first-onset and chronic orofacial pain, while gastroesophageal reflux disease and polycystic ovary syndrome have also shown relevant associations with temporomandibular disorders [18]. This broader pattern of multimorbidity is particularly relevant in FM and further supports the investigation of craniofacial processing in this population.

Despite growing evidence of alterations in both body image and body schema among individuals with chronic pain, the relationship between these constructs remains poorly understood in FM. Previous research has focused predominantly on limb-related laterality discrimination, and little is known about whether these alterations extend to craniofacial representations such as the mandible and tongue. Understanding whether disturbances in the sensorimotor representation of the body are associated with body image, psychological distress, and cognitive–affective aspects of pain may provide new insights into the mechanisms underlying the disorder.

Therefore, the primary objective was to compare craniofacial laterality discrimination, body esteem, state anxiety, and kinesiophobia between women with fibromyalgia and healthy controls. The secondary objective was to examine the associations between craniofacial laterality discrimination performance and body esteem, state anxiety, kinesiophobia, pain intensity, and fibromyalgia impact in women with fibromyalgia.

## 2. Materials and Methods

### 2.1. Study Design

A cross-sectional study was conducted and reported in accordance with the Strengthening the Reporting of Observational Studies in Epidemiology (STROBE) statement [19]. The completed checklist is provided as Supplementary Material.

### 2.2. Participants

Thirty women diagnosed with fibromyalgia were recruited from the fibromyalgia association AFINSYFACRO (Móstoles, Spain). All participants had an established diagnosis of fibromyalgia made by medical specialists according to the 2016 revised American College of Rheumatology diagnostic criteria and received medical follow-up within the context of the patient association. The diagnosis was not independently re-established by the research team at study enrollment. Individual Widespread Pain Index and Symptom Severity Scale scores and disease duration were not collected as study variables. Thirty healthy women were recruited as a control group from the University of Castilla-La Mancha (Toledo, Spain). Control participants were recruited from a different setting to minimize the likelihood of personal or social relationship with members of the fibromyalgia association. Participants were recruited and assessed between December 2025 and May 2026. Participants were enrolled through convenience sampling according to their availability and fulfillment of the eligibility criteria during the recruitment period. Eligible participants were adult women (≥18 years). No upper age limit was prespecified, and participants were not individually matched by age. Age was subsequently compared between groups as a baseline characteristic.

The study protocol was approved by the Ethical Review Board of Rey Juan Carlos University (approval number: 2605202012920) and was conducted in accordance with the principles of the Declaration of Helsinki. All participants provided written informed consent prior to participation.

The inclusion criteria for the FM group were: (1) an established diagnosis of fibromyalgia made by a medical specialist according to the 2016 revised American College of Rheumatology diagnostic criteria; (2) pain duration greater than 12 weeks; and (3) ability to speak and understand Spanish. Participants were excluded if they presented cognitive impairment preventing them from understanding or completing the study procedures or if they had previously participated in studies involving laterality recognition tasks.

The inclusion criteria for the control group were: (1) absence of pain at the time of assessment (NPRS = 0); (2) no current temporomandibular symptoms or orofacial pain and no previous diagnosis of temporomandibular disorder; (3) no cognitive problems that could interfere with understanding or completing the study procedures; (4) no current use of analgesic medication; and (5) ability to speak and understand Spanish. Control participants were excluded if they had experienced any musculoskeletal pain episode during the previous week or had a diagnosed rheumatological condition.

### 2.3. Pain and Clinical Status

Pain intensity and clinical status were assessed using self-administered questionnaires. Pain intensity was measured on a numeric pain rating scale (NPRS). NPRS is commonly used to assess self-reported pain in people with chronic pain. It ranges from 0, meaning no pain, to 10, meaning the worst possible pain. Scores of 1–3 indicate mild pain, 4–6 moderate pain, and 7–10 severe pain. The NPRS is considered a simple, reliable, and sensitive measure for both clinical practice and research [20].

The Spanish version of the Fibromyalgia Impact Questionnaire (FIQ) was used to assess the overall impact of fibromyalgia on daily functioning and health status. The Spanish version has demonstrated satisfactory reliability, validity, and responsiveness in individuals with fibromyalgia [21]. These outcomes were only assessed in the fibromyalgia group.

### 2.4. Anxiety

Anxiety was assessed using the State–Trait Anxiety Inventory (STAI). This questionnaire comprises two subscales: State Anxiety (S-Anxiety) and Trait Anxiety (T-Anxiety), each consisting of 20 items, for a total of 40 items. Only the State Anxiety subscale was administered in the present study. Responses are rated on a 4-point Likert scale ranging from 0 (“not at all”) to 3 (“very much so”). Total scores are calculated by summing the item scores after reversing the negatively worded items, with higher scores indicating greater levels of anxiety. The State Anxiety subscale assesses the respondent’s current level of anxiety by asking how they feel “right now.” It evaluates subjective feelings of apprehension, tension, nervousness, worry, and autonomic nervous system activation. The Spanish version of the State Anxiety subscale has demonstrated excellent internal consistency, with a Cronbach’s alpha coefficient of 0.94 [22].

### 2.5. Fear-Related Movement (Kinesiophobia)

Kinesiophobia was assessed using the Spanish version of the Tampa Scale for Kinesiophobia (TSK-11). The TSK-11 is an 11-item self-administered questionnaire designed to evaluate fear of movement and fear of reinjury associated with physical activity. Each item is scored on a 4-point Likert scale ranging from 1 (“strongly disagree”) to 4 (“strongly agree”), yielding a total score between 11 and 44 points. Higher scores indicate greater levels of kinesiophobia and fear-related avoidance beliefs toward movement and physical activity [23]. The Spanish version of the TSK-11 has demonstrated adequate psychometric properties in patients with chronic pain, showing good internal consistency (Cronbach’s α = 0.79) and supporting its use as a reliable measure of kinesiophobia in this population [24].

### 2.6. Body Esteem (BES)

Body esteem was assessed using the Body Esteem Scale (BES). This questionnaire consists of 35 items that evaluate the emotions elicited by different body parts and physical characteristics. Responses are rated on a 5-point Likert scale ranging from 1 (“strong negative feelings”) to 5 (“strong positive feelings”), with intermediate categories representing moderate negative feelings, neutral feelings, and moderate positive feelings. Total scores range from 35 to 175, with higher scores indicating more positive body esteem. The Spanish version of the BES has demonstrated excellent internal consistency, with a Cronbach’s alpha coefficient of 0.906 in women. In addition, the instrument showed high test–retest reliability, with an intraclass correlation coefficient of 0.87 (95% CI: 0.82–0.91) [25].

### 2.7. Left/Right Judgment Test (LRJT)

Craniofacial laterality discrimination was evaluated using a computerized left–right judgment task. Participants completed separate mandibular and lingual conditions, each comprising 30 images representing left- or right-sided positions. The stimuli were presented through the Cognitive Alpha 3.2 application on a Samsung Galaxy Tab S10 FE (Samsung Electronics Co., Ltd., Suwon, Republic of Korea). tablet with a 10.9-inch screen [26].

For each condition, the number of left- and right-sided stimuli, image viewpoints, degrees of rotation, and stimulus order were randomly determined by the application software. The sequence of the mandibular and lingual conditions was also randomized. Both the fibromyalgia and healthy control groups completed the tasks under the same standardized conditions.

Participants responded by touching the corresponding left or right button on the tablet screen and were instructed to determine the represented side as rapidly and accurately as possible. Each image remained on screen until a response was given or for a maximum of 10 s. Reaction time was calculated across both correct and incorrect trials. If no response was provided within 10 s, the trial was classified as incorrect and contributed to the reaction-time calculation. Mean reaction time, expressed in seconds, and recognition accuracy, expressed as the percentage of correct responses across the 30 trials, were retained for analysis.

Before the assessment, participants completed a familiarization round consisting of five practice images. They were instructed to remain still throughout the task to reduce the use of overt movement-based strategies. Hand dominance was not recorded. Participants who routinely used glasses or contact lenses continued to use their habitual visual correction during testing. The tasks were administered and supervised by the investigators, who recorded the final results generated by the application.

Cognitive was specifically developed to assess mandibular and lingual laterality discrimination in accordance with established laterality judgment paradigms. The stimulus images are proprietary and protected by copyright, and the application license does not permit their redistribution or publication; therefore, representative stimuli could not be provided as Supplementary Material.

### 2.8. Bias

To reduce measurement bias, all participants received standardized instructions and completed the laterality tasks under the same assessment conditions. The assessors did not have a previous relationship with the participants. Nevertheless, participants were aware of their clinical status, and blinding of participants and assessors to group allocation was not feasible.

### 2.9. Data Analysis

All statistical analyses were performed using IBM SPSS Statistics for Windows, version 31.0 (IBM Corp., Armonk, NY, USA). The normality of continuous variables was assessed using the Shapiro–Wilk test to guide the selection of parametric or non-parametric procedures. Descriptive data are presented as mean values and standard deviations. Women with FM and healthy controls were compared in terms of demographic characteristics, state anxiety, kinesiophobia, body esteem, and mandibular and lingual laterality discrimination outcomes. Independent-samples *t*-tests were used when the data followed a normal distribution, whereas Mann–Whitney U tests were applied to variables that did not meet the assumption of normality. For parametric comparisons, Cohen’s d was calculated to quantify the magnitude of between-group differences, with 95% confidence intervals reported where available and values of 0.20, 0.50, and 0.80 interpreted as small, moderate, and large effects, respectively. For non-parametric comparisons, effect size r was calculated from the standardized Mann–Whitney test statistic, with values of 0.10, 0.30, and 0.50 interpreted as small, moderate, and large effects, respectively. Effect sizes were reported for all between-group comparisons to provide information on the magnitude of the observed differences independently of statistical significance. No single craniofacial laterality outcome was prespecified as the primary endpoint. To account for multiple comparisons, the Holm procedure was applied separately to the four craniofacial laterality outcomes and to the three psychological/body-related outcomes. Because the application output available for analysis consisted of participant-level summary measures for each condition, group comparisons were performed using mean reaction time and percentage accuracy. Trial-level responses and stimulus identifiers were not retained, precluding mixed-effects modelling of participant- and stimulus-level variability. Within the FM group, associations between mandibular and lingual laterality discrimination outcomes and psychological and clinical variables were examined using Spearman’s rank correlation coefficients. The variables included body esteem, state anxiety, kinesiophobia, pain intensity, and fibromyalgia impact. Correlation coefficients were interpreted as weak when below 0.30, moderate between 0.30 and 0.59, and strong when equal to or greater than 0.60. All analyses were two-sided, and statistical significance was established at *p* < 0.05. No formal a priori sample-size calculation was performed. The sample size was determined by the number of eligible participants recruited during the predefined data-collection period. Given the sample available for the within-group correlation analyses, analyses addressing the secondary objective were considered exploratory. Because relatively low accuracy was observed in some participants, additional exploratory analyses examined performance relative to the 50% chance level. The number of participants with mean accuracy at or below 50% was also determined, and sensitivity analyses were performed after excluding these participants.

## 3. Results

### 3.1. Sociodemographic Characteristics and Clinical Status

A total of 32 women with FM were initially considered for participation. Two eligible women were unable to attend the scheduled assessment for personal reasons and were not evaluated. All healthy controls attended and completed the scheduled assessment. The final sample comprised 60 participants (30 women with FM and 30 healthy controls). All included participants completed all study assessments, and no missing data were recorded for the variables analyzed. No significant between-group differences were observed in demographic characteristics, including age (48.07 ± 8.19 vs. 47.97 ± 7.60 years; *p* = 0.961), body weight (68.10 ± 13.54 vs. 67.80 ± 11.96 kg; *p* = 0.950), or height (164.07 ± 7.79 vs. 165.33 ± 7.16 cm; *p* = 0.520).

Regarding clinical characteristics, women with FM reported a mean pain intensity of 7.40 ± 1.57 points on the NPRS and a mean FIQ score of 68.30 ± 13.30 points. The observed age range was 32–65 years in the FM group and 31–61 years in the healthy control group. Results are presented in Table 1.

### 3.2. Psychological Characteristics and Body Esteem

Women with FM showed significantly higher state anxiety than healthy controls (31.40 ± 8.32 vs. 16.27 ± 7.49; *p* < 0.001; Cohen’s d = 1.91) and higher kinesiophobia (27.53 ± 5.15 vs. 21.27 ± 4.24; *p* < 0.001; Cohen’s d = 1.33). In contrast, body esteem was significantly lower in women with FM than in healthy controls (86.43 ± 20.29 vs. 125.20 ± 23.86; *p* < 0.001; Cohen’s d = −1.75. All between-group differences remained statistically significant after Holm correction and showed large effect sizes. Results are presented in Table 2.

### 3.3. Craniofacial Laterality Discrimination

In the unadjusted analyses, women with FM showed lower mandibular laterality accuracy than healthy controls (49.07 ± 15.35% vs. 57.95 ± 16.45%; *p* = 0.035; Cohen’s d = −0.56) and longer lingual reaction times (3.04 ± 0.92 vs. 2.54 ± 0.96 s; *p* = 0.028; r = 0.28). However, neither difference remained statistically significant after Holm correction for the four craniofacial laterality outcomes (adjusted *p* = 0.112 for both comparisons). No significant between-group differences were observed for mandibular reaction time (*p* = 0.284; adjusted *p* = 0.284) or lingual accuracy (*p* = 0.074; adjusted *p* = 0.148). Results are presented in Table 3.

Additional exploratory analyses were conducted to examine performance relative to the 50% chance level. For mandibular laterality, 17 of 30 women with FM and 10 of 30 healthy controls showed mean accuracy ≤50%. For lingual laterality, this occurred in 13 of 30 women with FM and 8 of 30 controls. At the group level, accuracy in healthy controls was significantly above 50% for both mandibular and lingual tasks, whereas accuracy in the FM group did not significantly differ from the 50% level. In sensitivity analyses excluding participants with accuracy ≤50%, 13 women with FM and 20 healthy controls remained for the mandibular task, and 17 women with FM and 22 healthy controls remained for the lingual task. No statistically significant between-group differences were observed after these exclusions for mandibular accuracy (U = 82.5, *p* = 0.083) or lingual accuracy (U = 131.0, *p* = 0.116).

### 3.4. Associations with Psychological and Clinical Variables

No statistically significant correlations were found between mandibular or lingual laterality performance and state anxiety, kinesiophobia, body esteem, pain intensity, or fibromyalgia impact in women with FM. Correlation coefficients were generally weak, ranging from ρ = −0.300 to ρ = 0.354, with all *p* values > 0.05.

The strongest association was observed between fibromyalgia impact and mandibular reaction time (ρ = 0.354, *p* = 0.055), although it did not reach statistical significance. Kinesiophobia also showed a weak-to-moderate positive association with mandibular reaction time (ρ = 0.306, *p* = 0.100), while body esteem showed a negative association with lingual reaction time (ρ = −0.300, *p* = 0.108). Results are presented in Table 4.

## 4. Discussion

The present study investigated differences in craniofacial laterality discrimination, body esteem, state anxiety, and kinesiophobia between women with FM and healthy controls, as well as the associations between laterality performance and psychological and clinical variables within the FM group. In the unadjusted analyses, women with FM showed poorer mandibular recognition accuracy and longer reaction times during the lingual laterality task; however, these differences did not remain statistically significant after correction for multiple comparisons. No between-group differences were observed in mandibular reaction time or lingual accuracy. In contrast, women with FM reported markedly higher levels of state anxiety and kinesiophobia and lower body esteem than healthy controls, and these differences remained statistically significant after multiplicity adjustment. Craniofacial laterality performance was not significantly associated with body esteem, anxiety, kinesiophobia, pain intensity, or fibromyalgia impact. The novelty of the present work lies in extending the study of laterality discrimination beyond the limbs to craniofacial regions and in examining these outcomes alongside body esteem and psychological factors. Overall, the observed pattern suggests that craniofacial laterality performance warrants further investigation in FM, although the present exploratory association analyses do not support firm conclusions regarding their relationship with emotional distress, fear of movement, negative body evaluation, pain intensity, or overall disease impact. The present findings are partially consistent with those reported by Martínez et al. [10], who observed both lower accuracy and longer response times during laterality discrimination in individuals with FM compared with pain-free controls. In the present study, women with FM showed reduced mandibular recognition accuracy and longer lingual response times in the unadjusted analyses; however, these differences did not remain statistically significant after correction for multiple comparisons, while no significant differences were found for mandibular reaction time or lingual accuracy. These findings may indicate a pattern of altered craniofacial laterality performance in FM that warrants further investigation, although the present results should be interpreted cautiously. Differences in stimulus characteristics, task difficulty, and the body parts examined may partly explain the more selective pattern observed in our sample.

The present findings should also be considered within the broader literature on laterality discrimination in chronic musculoskeletal pain. A systematic review and meta-analysis by Breckenridge et al. [27] reported altered LRJT performance across several chronic musculoskeletal pain populations, although the consistency and magnitude of these alterations varied according to the clinical condition, with more consistent findings in peripheral and facial pain than in axial pain conditions. This heterogeneity suggests that impaired laterality-task performance should not be regarded as a uniform feature of chronic pain or attributed to a single underlying mechanism. Evidence from FM further supports this heterogeneous interpretation. Previous work found associations between laterality discrimination performance and conditioned pain modulation, and a subsequent secondary analysis showed poorer LRJT performance among women with FM classified as non-responders to conditioned pain modulation compared with responders [28]. These findings suggest that laterality-task performance may vary according to pain-modulation characteristics and reinforce the need to avoid interpreting the absence of significant associations in the present sample as evidence that craniofacial laterality performance is independent of clinical or pain-processing factors. It is also important to distinguish LRJT performance from the theoretical constructs proposed to contribute to it. Accuracy and reaction time represent behavioral task outcomes, whereas body schema, body representation, and implicit motor imagery are related but distinct theoretical constructs that cannot be directly inferred from LRJT performance alone.

The psychological findings are consistent with those reported in a previous study [29], in which women with FM showed significantly higher levels of anxiety, depression, pain catastrophizing, and kinesiophobia than healthy controls. Despite this marked psychological burden, no significant associations were identified between these variables and limb laterality discrimination performance. A similar pattern was observed in the present study, as women with FM reported greater state anxiety and kinesiophobia, but neither variable was significantly associated with mandibular or lingual laterality outcomes. These convergent findings indicate that psychological distress is an important feature of the clinical profile of FM, although its relationship with laterality performance remains uncertain.

Previous research in women with chronic temporomandibular disorders also reported poorer mandibular and lingual laterality discrimination and slower responses than in asymptomatic controls [26]. However, that study primarily focused on craniofacial pain and disability and did not directly assess anxiety, kinesiophobia, or other psychological variables. Therefore, the present study extends these findings by examining craniofacial laterality performance together with emotional and body-related factors in women with FM. Despite the marked differences in anxiety, kinesiophobia, and body esteem between groups, these variables were not significantly associated with laterality performance, although the exploratory nature of these analyses precludes firm conclusions regarding the relationship between psychological distress and craniofacial laterality performance. The craniofacial focus of the present study should also be considered in the context of the substantial prevalence and comorbidity of orofacial pain. Recent evidence indicates that myofascial and temporomandibular joint-related pain affect a meaningful proportion of the population and that orofacial pain may overlap with chronic pain conditions such as irritable bowel syndrome, gastroesophageal reflux disease, and polycystic ovary syndrome [17,18]. Although these conditions were not systematically characterized in the present FM sample, their potential influence on craniofacial laterality performance cannot be excluded.

Similar findings have been reported in individuals with recurrent neck pain, who showed poorer laterality judgment accuracy than pain-free controls [30]. Although fear avoidance beliefs were assessed in that study, they were not directly associated with laterality performance. Instead, laterality accuracy was related to neck disability, while fear-avoidance beliefs were more closely associated with disability and tactile discrimination. This pattern is consistent with the present results, suggesting that psychological factors may contribute to the broader clinical burden of chronic pain without necessarily being directly related to performance in implicit laterality tasks.

Another study involving patients with complex regional pain syndrome also considered depressive symptoms when examining mental rotation of facial expressions [31]. Although patients showed substantially higher BDI scores than healthy controls, depression was not significantly associated with reaction time and did not account for the between-group differences in task performance. This finding is consistent with the present results, in which state anxiety and kinesiophobia were markedly elevated in women with FM but were not significantly related to mandibular or lingual laterality performance. Together, these findings suggest that psychological distress may coexist with altered body-related processing, although the present exploratory analyses do not allow firm conclusions regarding whether psychological factors directly influence laterality performance. Similar results have been reported in patients with chronic whiplash-associated disorders, in whom post-traumatic stress symptoms were assessed using the Post-traumatic Stress Diagnostic Scale [32]. Neither the severity of post-traumatic stress symptoms nor pain and disability measures were significantly associated with neck laterality performance. Although laterality judgments were not impaired in that population, these findings further suggest that psychological distress may not consistently be associated with poorer performance on implicit body-related tasks.

### 4.1. Clinical Implications

From a clinical perspective, the present findings should not be interpreted as supporting the routine use of craniofacial laterality discrimination tasks in women with FM. Given the cross-sectional design, the relatively small sample, the absence of statistically significant craniofacial differences after correction for multiple comparisons, the relatively low accuracy observed in a proportion of participants, and the lack of established clinically meaningful thresholds, these tasks should currently be considered experimental research measures requiring further validation. The lack of statistically significant associations with anxiety, kinesiophobia, body esteem, pain intensity, and fibromyalgia impact should also be interpreted cautiously given the exploratory nature of these analyses. Accordingly, assessment and treatment of women with FM should remain multidimensional and should not assume that craniofacial laterality performance reflects a single underlying mechanism. Moreover, performance on these tasks should not be interpreted as a direct measure of implicit motor imagery or cortical body representation. Further research is needed to establish their test–retest reliability, stimulus-related performance characteristics, clinically meaningful thresholds, and potential clinical utility before their incorporation into routine clinical assessment can be recommended.

### 4.2. Limitations

Several limitations should be considered when interpreting these findings. First, the cross-sectional design prevents causal inferences regarding the relationships between craniofacial laterality performance and psychological or clinical variables. Second, the relatively small convenience sample, particularly the 30 women included in the correlation analyses, limited the ability to detect small-to-moderate associations. Therefore, non-significant correlations should be interpreted cautiously and should not be considered evidence of the absence of such relationships. The inclusion of women only and the recruitment of patients and controls from different settings also restrict the generalizability of the results and may have introduced selection bias. Although fibromyalgia diagnosis had been established by medical specialists according to ACR criteria, no independent diagnostic reassessment was performed and WPI/SSS scores and disease duration were not collected. Potentially relevant factors such as medication use, duration of FM, cognitive status, temporomandibular symptoms and orofacial pain were not fully controlled. Questionnaire-based measures may be subject to self-report and recall bias. In addition, performance on the computerized laterality tasks may have been influenced by individual differences in attention, familiarity with digital devices, and motor-response variability. Although all participants completed the tasks under standardized conditions, the mandibular and lingual laterality paradigms have limited external validation, and their psychometric properties require further investigation.

### 4.3. Future Research Lines

Future research should confirm these findings in larger and more heterogeneous samples and examine whether craniofacial laterality performance differs according to fibromyalgia severity, orofacial pain, temporomandibular symptoms, medication use, or psychological profile. Longitudinal studies are also needed to determine whether changes in laterality performance accompany variations in pain, disability, or body-related perceptions over time. In addition, further validation of mandibular and lingual laterality tasks is warranted, including the analysis of stimulus difficulty, test–retest reliability, and clinically meaningful cut-off values. Future studies should also incorporate general reaction-time or non-body mental-rotation control tasks and examine stimulus-level effects such as rotation angle or biomechanical plausibility to better characterize the cognitive mechanisms underlying craniofacial laterality performance. Future studies should also systematically assess orofacial pain, temporomandibular disorders, and headache using validated criteria, preferably ICOP and DC/TMD, to examine their potential influence on craniofacial laterality performance. Finally, clinical trials should investigate whether interventions targeting implicit motor imagery, graded motor imagery, or other sensorimotor approaches can modify craniofacial laterality performance and whether such changes are associated with improvements in pain, function, or quality of life. 

## 5. Conclusions

Women with fibromyalgia showed marked differences in state anxiety, kinesiophobia, and body esteem compared with healthy controls. Although unadjusted analyses suggested differences in craniofacial laterality performance, these findings did not remain statistically significant after correction for multiple comparisons. Exploratory correlation analyses did not identify statistically significant associations between laterality performance and psychological variables, pain intensity, or fibromyalgia impact.

## Figures and Tables

**Table 1:** Sociodemographic Characteristics and Clinical Status. Data are presented as mean ± standard deviation. Between-group comparisons of age, body weight, and height were performed using independent-samples Student’s *t*-tests. Pain intensity and fibromyalgia impact were assessed only in the fibromyalgia group. NPRS = Numeric Pain Rating Scale; FIQ = Fibromyalgia Impact Questionnaire.

Variable	Fibromyalgia (n = 30)	Healthy Controls (n = 30)	*p* Value
Sociodemographic characteristics			
Age (years)	48.07 ± 8.19	47.97 ± 7.60	0.961
Body weight (kg)	68.10 ± 13.54	67.80 ± 11.96	0.950
Height (cm)	164.07 ± 7.79	165.33 ± 7.16	0.520
Clinical status			
Pain intensity, NPRS	7.40 ± 1.57	Not applicable	—
Fibromyalgia impact, FIQ	68.30 ± 13.30	Not applicable	—

**Table 2:** Comparison of state anxiety, kinesiophobia, and body esteem between women with fibromyalgia and healthy controls. Data are presented as mean ± standard deviation. Independent-samples *t*-tests were used for group comparisons. Raw *p* values and Holm-adjusted *p* values are reported. Positive effect sizes indicate higher scores in the fibromyalgia group, whereas negative values indicate lower scores. STAI-State = State Anxiety subscale of the State–Trait Anxiety Inventory; TSK-11 = 11-item Tampa Scale for Kinesiophobia; BES = Body Esteem Scale.

Variable	Fibromyalgia (n = 30)	Healthy Controls (n = 30)	Statistical Test	*p* Value	Holm-Adjusted *p*	Effect Size (95% CI)
STAI-State	31.40 ± 8.32	16.27 ± 7.49	t Student	<0.001	<0.001	Cohen’s *d* = 1.91; 95% CI [1.30, 2.53]
TSK-11	27.53 ± 5.15	21.27 ± 4.24	t Student	<0.001	<0.001	Cohen’s *d* = 1.33; 95% CI [0.77, 1.89]
BES	86.43 ± 20.29	125.20 ± 23.86	t Student	<0.001	<0.001	Cohen’s *d* = −1.75; 95% CI [−2.35, −1.15]

**Table 3:** Comparison of mandibular and lingual laterality discrimination performance between women with fibromyalgia and healthy controls. Data are presented as mean ± standard deviation. An independent-samples *t*-test was used for mandibular accuracy, whereas Mann–Whitney U tests were used for mandibular reaction time, lingual accuracy, and lingual reaction time according to data distribution. Raw *p* values and Holm-adjusted *p* values are reported. Cohen’s d was reported for the parametric comparison and effect size r for the non-parametric comparisons. Effect sizes of 0.20, 0.50, and 0.80 for Cohen’s d and 0.10, 0.30, and 0.50 for r were interpreted as small, moderate, and large, respectively.

Variable	Fibromyalgia (n = 30)	Healthy Controls (n = 30)	Statistical Test	*p* Value	Holm-Adjusted *p*	Effect Size (95% CI)
Mandibular accuracy (%)	49.07 ± 15.35	57.95 ± 16.45	t Student	0.035	0.112	Cohen’s *d* = −0.56; 95% CI [−1.07, −0.04]
Mandibular reaction time (s)	3.08 ± 0.80	2.92 ± 0.84	U Mann–Whitney	0.284	0.284	r = 0.14
Lingual accuracy (%)	53.52 ± 21.47	62.63 ± 20.80	U Mann–Whitney	0.074	0.148	r = 0.23
Lingual reaction time (s)	3.04 ± 0.92	2.54 ± 0.96	U Mann–Whitney	0.028	0.112	r = 0.28

**Table 4:** Spearman correlation coefficients between mandibular and lingual laterality discrimination outcomes and psychological and clinical variables in women with fibromyalgia. Values are presented as Spearman’s ρ (*p* value). No statistically significant associations were identified. STAI-State = State Anxiety subscale of the State–Trait Anxiety Inventory; TSK-11 = 11-item Tampa Scale for Kinesiophobia; BES = Body Esteem Scale; NPRS = Numeric Pain Rating Scale; FIQ = Fibromyalgia Impact Questionnaire.

Variable	Mandibular Reaction Time ρ (*p*)	Mandibular Accuracy ρ (*p*)	Lingual Reaction Time ρ (*p*)	Lingual Accuracy ρ (*p*)
State anxiety, STAI-State	0.094 (0.621)	0.051 (0.788)	0.196 (0.299)	−0.089 (0.640)
Kinesiophobia, TSK-11	0.306 (0.100)	−0.251 (0.181)	0.229 (0.224)	−0.260 (0.165)
Body esteem, BES	−0.201 (0.286)	0.244 (0.193)	−0.300 (0.108)	0.178 (0.346)
Pain intensity, NPRS	0.270 (0.149)	−0.017 (0.927)	0.107 (0.572)	−0.094 (0.620)
Fibromyalgia impact, FIQ	0.354 (0.055)	−0.087 (0.648)	0.213 (0.257)	−0.099 (0.602)

## Data Availability

De-identified participant-level data supporting the findings of this study are available from the corresponding author upon reasonable request. Data are unavailable due to privacy or ethical restrictions.

## Supplementary Materials

The following supporting information can be downloaded at: https://www.mdpi.com/article/10.3390/healthcare14162636/s1, STROBE Statement—Checklist.
