# Supplementary material for: Craniofacial Laterality Discrimination and Psychological Factors in Women with Fibromyalgia: A Cross-Sectional Study
Source: Healthcare (Basel). 2026 Aug 20;14(16):2636. doi: 10.3390/healthcare14162636 (PMC13511799; doi:10.3390/healthcare14162636)
Supplement: Supplementary file 1 [file healthcare-14-02636-s001.zip › healthcare-4494796-supplementary.pdf]

STROBE Statement—Checklist

	Item No	Recommendation
**Title and abstract**	1	(*a*) Indicate the study's design with a commonly used term in the title or the abstract: **Reported on page 1**
		(*b*) Provide in the abstract an informative and balanced summary of what was done and what was found. **Reported on page 1**
**Introduction**		
Background/rationale	2	Explain the scientific background and rationale for the investigation being reported. **Reported on page 2-3**
Objectives	3	State specific objectives, including any prespecified hypotheses. **Reported on page 3**
**Methods**		
Study design	4	Present key elements of study design early in the paper. **Reported on page 3**
Setting	5	Describe the setting, locations, and relevant dates, including periods of recruitment, exposure, follow-up, and data collection. **Reported on page 3**
Participants	6	(*a*) Give the eligibility criteria, and the sources and methods of selection of participants. **Reported on page 3-4**
Variables	7	Clearly define all outcomes, exposures, predictors, potential confounders, and effect modifiers. Give diagnostic criteria, if applicable. **Reported on page 3-5.**
Data sources/ measurement	8	For each variable of interest, give sources of data and details of methods of assessment (measurement). Describe comparability of assessment methods if there is more than one group. **Reported on page 5-6**
Bias	9	Describe any efforts to address potential sources of bias. **Reported on page 6**
Study size	10	Explain how the study size was arrived at. **Reported on page 6**
Quantitative variables	11	Explain how quantitative variables were handled in the analyses. If applicable, describe which groupings were chosen and why. **Reported on page 6**
Statistical methods	12	(*a*) Describe all statistical methods, including those used to control for confounding. **Reported on page 6**
		(*b*) Describe any methods used to examine subgroups and interactions. **Not applicable**
		(*c*) Explain how missing data were addressed. **Reported on page 7**
		(*d*) If applicable, describe analytical methods taking account of sampling strategy. **Not applicable – convenience sampling was used, and no sampling weights or complex survey design methods were required.**
		(*e*) Describe any sensitivity analyses. **Reported on page 6, 8-9**
**Results**		
Participants	13	(a) Report numbers of individuals at each stage of study—eg numbers potentially eligible, examined for eligibility, confirmed eligible, included in the study, completing follow-up, and analysed. **Reported on page 6-7**
		(b) Give reasons for non-participation at each stage. **Reported on page 6**
		(c) Consider use of a flow diagram. **Not applicable; participant flow reported in text, pp. 6–7**
Descriptive data	14	(a) Give characteristics of study participants (eg demographic, clinical, social) and information on exposures and potential confounders. . **Reported on page 7, table 1.**
		(b) Indicate number of participants with missing data for each variable of interest. . **Reported on page 7**

Outcome data	15	Report numbers of outcome events or summary measures. **Reported on page 7-8. Tables 2-4**
Main results	16	(*a*) Give unadjusted estimates and, if applicable, confounder-adjusted estimates and their precision (eg, 95% confidence interval). Make clear which confounders were adjusted for and why they were included. **Reported on page 7-8. Tables 2-3**
		(*b*) Report category boundaries when continuous variables were categorized. **Report category boundaries when continuous variables were categorized. Reported on pages 8–9.**
		(*c*) If relevant, consider translating estimates of relative risk into absolute risk for a meaningful time period. **Not applicable**
Other analyses	17	Report other analyses done—eg analyses of subgroups and interactions, and sensitivity analyses. . **Reported on page 8-9.**
**Discussion**		
Key results	18	Summarise key results with reference to study objectives. **Reported on page 9-10**
Limitations	19	Discuss limitations of the study, taking into account sources of potential bias or imprecision. Discuss both direction and magnitude of any potential bias. . **Reported on page 12**
Interpretation	20	Give a cautious overall interpretation of results considering objectives, limitations, multiplicity of analyses, results from similar studies, and other relevant evidence. . **Reported on page 9-12**
Generalisability	21	Discuss the generalisability (external validity) of the study results. **Reported on page 12**
**Other information**		
Funding	22	Give the source of funding and the role of the funders for the present study and, if applicable, for the original study on which the present article is based. . **Reported on page 13**
